# Tight Regulation of Srs2 Helicase Activity Is Crucial for Proper Functioning of DNA Repair Mechanisms

**DOI:** 10.1534/g3.118.200181

**Published:** 2018-03-12

**Authors:** Alex Bronstein, Shay Bramson, Keren Shemesh, Batia Liefshitz, Martin Kupiec

**Affiliations:** Department of Molecular Microbiology and Biotechnology, Tel Aviv University, Ramat Aviv 69978, Israel

**Keywords:** helicases, homologous recombination, Srs2, yeast

## Abstract

Proper DNA damage repair is one of the most vital and fundamental functions of every cell. Several different repair mechanisms exist to deal with various types of DNA damage, in various stages of the cell cycle and under different conditions. Homologous recombination is one of the most important repair mechanisms in all organisms. Srs2, a regulator of homologous recombination, is a DNA helicase involved in DNA repair, cell cycle progression and genome integrity. Srs2 can remove Rad51 from ssDNA, and is thought to inhibit unscheduled recombination. However, Srs2 has to be precisely regulated, as failure to do so is toxic and can lead to cell death. We noticed that a very slight elevation of the levels of Srs2 (by addition of a single extra copy of the *SRS2* gene) leads to hyper-sensitivity of yeast cells to methyl methanesulfonate (MMS, a DNA damaging agent). This effect is seen in haploid, but not in diploid, cells. We analyzed the mechanism that controls haploid/diploid sensitivity and arrived to the conclusion that the sensitivity requires the activity of *RAD59* and *RDH54*, whose expression in diploid cells is repressed. We carried out a mutational analysis of Srs2 to determine the regions of the protein required for the sensitization to genotoxins. Interestingly, Srs2 needs the HR machinery and its helicase activity for its toxicity, but does not need to dismantle Rad51. Our work underscores the tight regulation that is required on the levels of Srs2 activity, and the fact that Srs2 helicase activity plays a more central role in DNA repair than the ability of Srs2 to dismantle Rad51 filaments.

DNA is constantly exposed to damaging agents; these can be either exogenous (radiation, chemicals) or endogenous, like natural products of cellular metabolism. The probability of occurrence of damage to the genetic material increases during DNA replication, when the DNA has to be unpacked and exposed. The activity of the DNA polymerases may be disrupted by the presence of DNA secondary structures, bound proteins or lesions; this may lead to stalling or even collapse of replication forks (dmowski and fijalkowska 2017). In response, cellular mechanisms are activated and arrest cell cycle progression, induce DNA repair, and restore replication (kolodner *et al.* 2002; lue and yu 2017; palou *et al.* 2017). These response mechanisms act either to promote repair of the lesions or to allow their bypass, thus preventing them from being converted into fatal genomic rearrangements. The genetic pathways responsible for DNA repair and genome stability are highly conserved across species (boiteux and jinks-robertson 2013; gadaleta *et al.* 2016).

To deal with DNA damage during replication, cells have developed sophisticated mechanisms that overcome replication-blocking damage during S phase. Upon DNA damage and replication stalling, the DNA polymerase processivity clamp, PCNA, is modified by the addition of either ubiquitin or SUMO. Ubiquitylation of PCNA promotes two DNA damage tolerance (DDT) pathways of damage bypass: a) the error-prone pathway, by mono-ubiquitylation of PCNA at a lysine residue, K164, by the Rad6/Rad18 complex (bailly *et al.* 1994), thus promoting the switch between replicative and translesion-specific polymerases ; or b) the error-free pathway, by extending the mono-ubiqutin to a poly-ubiquitin with the help of the Ubc13/Mms2 E2 in cooperation with the Rad5 E3 (hofmann and pickart 1999; hoege *et al.* 2002). This initiates a bypass mechanism that is not entirely understood but probably entails copying information from the sister chromatid. In addition to those repair mechanisms, cells also utilize the homologous recombination (HR) repair pathway. (prakash 1981; zhang and lawrence 2005; gangavarapu *et al.* 2007). The HR reactions are catalyzed by members of the *RAD52* epistasis group (*RAD51*, *RAD52*, *RAD54*, *RAD55*, *RAD57*, *RAD59*). Yeast cells mutated for these genes are defective in the repair of DNA damage caused by ionizing radiation and methyl methane sulfonate (MMS), in mitotic and meiotic recombination, and in mating-type switching [as double-stranded break (DSB) repair intermediates are involved in these processes] (symington and gautier 2011; gao *et al.* 2014; adamczyk *et al.* 2016). Some of the group members are important for catalyzing only some of pathways mentioned above, while lacking a role in others.

Rad52 is required for most of the HR mediated DSB repair mechanisms, and hence its absence confers the most severe phenotype. Rad52, together with Rad55 and Rad57, plays a role in the loading of Rad51 onto DNA (gaines *et al.* 2015) ; in addition, it acts in annealing complementary strands of ssDNA (mortensen *et al.* 1996; shinohara *et al.* 1998; davis and symington 2001). *RAD59* encodes a protein that resembles a truncated version of Rad52 and also works in ssDNA annealing (petukhova *et al.* 1999; wu *et al.* 2006). The interaction of Rad52 with Rad59 is important for Rad51-independent DSB repair pathways, such as single-strand annealing (SSA), a form of direct-repeat recombination (DRR) (sugawara *et al.* 2000; davis and symington 2001; pannunzio *et al.* 2008).

Rad51 is also essential for the repair of DSBs. It binds to ssDNA and stimulates strand exchange with the donor DNA. Rad51 is the main player in the SDSA pathway, which can result in the nonreciprocal transfer of information between interacting DNA molecules (gene conversion, GC). It is also necessary for crossover formation, but does not play a role in SSA (symington and gautier 2011; boiteux and jinks-robertson 2013).

Srs2 plays a role in the regulation of HR. Srs2 is a DNA helicase that is able to unwind DNA substrates containing forks, flaps, D-loops, 3′ and 5′ single stranded DNA overhangs, blunt-end double stranded DNA substrates as well as Holliday junctions (van komen *et al.* 2003; marini and krejci 2012). Srs2 is also able to displace Rad51 from ssDNA (krejci *et al.* 2003; veaute *et al.* 2003; antony *et al.* 2009), and it is thus many times referred to as an “anti-recombinase”. Recently it was also shown that Srs2 is able to disrupt extended D-loops such as those created by Rad51 and Rad54 during SDSA repair (liu *et al.* 2017). SUMOylated PCNA recruits Srs2 to replication forks, where it appears to prevent unscheduled recombination events (papouli *et al.* 2005; pfander *et al.* 2005). However, depending on the assay used, Srs2 has been shown to promote (aylon *et al.* 2003) or prevent (schiestl *et al.* 1990) homologous recombination. Srs2 was shown to act in the promotion of SDSA and inhibition of crossover events (ira *et al.* 2003; robert *et al.* 2006; miura *et al.* 2013), as well as in SSA and break-induced replication (BIR) (sugawara *et al.* 2000; carter *et al.* 2009; ruiz *et al.* 2009). Moreover, Srs2 also has a yet ill-defined role in controlling cell cycle arrest and growth resumption following DNA damage (liberi *et al.* 2000; vaze *et al.* 2002).

Srs2 is an important guardian of genome stability, but if unregulated, it can generate HR intermediates causing DNA damage, blocked replication forks, or nucleoprotein complexes that can lead to cell cycle arrest and even cause cell death in certain genetic backgrounds (gangloff *et al.* 2000; León Ortiz *et al.* 2011). Therefore, it must be tightly regulated to execute its biochemical activities in a precise manner. In this work, we show that Srs2 is carefully regulated when cells deal with DNA damage. Even slight increases in the levels of Srs2 lead to inhibition of DNA repair. This negative effect of Srs2 over-activity requires the Rdh54 DNA translocase and Rad59, and is specific for haploid cells. Most importantly, we show that Srs2’s DNA helicase activity and not Rad51 dismantling activity is required for Srs2’s role in HR repair and in the creation of toxic intermediates.

## Materials and Methods

### Yeast strains

Unless mentioned otherwise, all yeast strains are derivatives of MK166 (liefshitz *et al.* 1995): MATa lys2:: Ty1Sup ade2-1(o) can1-100(o) ura3-52 leu2-3, 112 his3del200 trp1del901 HIS3:: lys2:: ura3 his4:: TRP1:: his4.

Standard Yeast Molecular genetics techniques were used to delete individual genes.

### Plasmids

Low overexpression of *SRS2* and *SRS2* mutants was obtained by introducing SRS2 under its natural promoter on the centromeric plasmids YCp50 or pRS316.

Strains and plasmids are listed in Table 1 and Table 2 respectively.

**Table 1:** Yeast strains used in this work

Name	Relevant genotype	Source
MK166 diploid	*MATa/MATα*	(liefshitz *et al.* 1995)
AB101	*MK166 MATa*	(liefshitz *et al.* 1995)
AB121	MK166 *MATa rad51*:: *LEU2*	(liefshitz *et al.* 1995)
AB124	MK166 *MATa rad52*:: *LEU2*	(liefshitz *et al.* 1995)
BL218	MK166 *MATa rad55*:: *LEU2*	(liefshitz *et al.* 1995)
BLY326	MK166 *MATa rad57*:: *LEU2*	(liefshitz *et al.* 1995)
MK118	MK166 *MATa rad59*:: *KanMX*	(jablonovich *et al.* 1999)
AB134	MK166 *MATa rad54*:: *KanMX*	This study
AB465	MK166 *MATa rdh54*:: *HygMX*	This study
AB456	MK166 *MATa nej1*:: *KanMX*	This study
BY4741	*MATa his3Δ1 leu2Δ0 met15Δ0 ura3Δ0*	Lab stock
BY4741 *Δddr2*	*MATa his3Δ1 leu2Δ0 met15Δ0 ura3Δ0 ddr2*::*KanMX*	Deletion library
BY4741 *Δfar1*	*MATa his3Δ1 leu2Δ0 met15Δ0 ura3Δ0 far1*::*KanMX*	Deletion library
BY4741 *Δfus3*	*MATa his3Δ1 leu2Δ0 met15Δ0 ura3Δ0 fus3*::*KanMX*	Deletion library
BY4741 *Δgpa1*	*MATa his3Δ1 leu2Δ0 met15Δ0 ura3Δ0 gpa1*::*KanMX*	Deletion library
BY4741 *Δamn1*	*MATa his3Δ1 leu2Δ0 met15Δ0 ura3Δ0 amn1*::*KanMX*	Deletion library
AB217	MK166 *MATa mrc1*::*natR*	This study
MK4193	MK166 *MATa rad24*::*KanMX*	This study
AB91	MK166 *MATa rad9*::*natR*	This study
AB155	MK166 *MATa pol30 -K127R,K164R*::*KanMX*	This study
OP1122	MK166 *MATa rad18*::*LEU2*	(liefshitz *et al.* 1998)
op890	MK166 *MATa rad5*::*KanMX*	(liefshitz *et al.* 1998)
AB171	MK166 *MATa pol30-K164R*::*KanMX*	This study
op883	MK166 *MATa srs2*::*KanMX*	(liefshitz *et al.* 1998)
OP1125	MK166 MATa *rad18*::*LEU2 srs2*::*KanMX*	(friedl *et al.* 2001)
AB234	MK166 *MATa rad5*::*KanMX srs2*::*KanMX*	(friedl *et al.* 2001)
AB270	MK166 *MATa pol30-K164R*::*KanMX srs2*::*KanMX*	This study
AB583	MK166 *MATa srs2*::*KanMX rad51*::*LEU2 rdh54*::*HygMX*	This study

**Table 2:** plasmids used in this work

Name	Relevant genotype	Source
p14H	*SRS2* in YCp50	(aboussekhra *et al.* 1989)
pCB115	*MAT* a/α plasmid	(liefshitz *et al.* 1995)
pAM28	*rad51*:: *LEU2* Disruptor	(liefshitz *et al.* 1995)
pSM20	*rad52*:: *LEU2* Disruptor	(liefshitz *et al.* 1995)
AB102	*SRS2* in pRS316	This study
AB123	*srs2-K41A* in pRS316	This study
AB095	*srs2ΔSIM* in pRS316	This study
AB177	*srs2Δ(875-902)-L844A* in pRS316	This study
SB1071	w.t *SRS2* in yEplac22	(saponaro *et al.* 2010)
SB1016	*srs2AV* in yEplac22	(saponaro *et al.* 2010)
SB1070	*srs2DE* in yEplac22	(saponaro *et al.* 2010)
SB1203	*srs2KR* in yEplac22	(saponaro *et al.* 2010)
SB1204	*srs2AVKR* in yEplac22	(saponaro *et al.* 2010)

### MMS sensitivity

Serial ten-fold dilutions of logarithmic yeast cells were spotted on fresh Synthetic Dextrose (SD)-complete (or SD lacking a specific amino acid to preserve the plasmid) plates with or without different concentrations of Methyl methane sulfonate (MMS; Sigma) and incubated at 30° for three days.

### Determination of recombination rates

Strain MK166 carries substrates that allow easy scoring of direct repeat recombination (DRR; His+ colonies) and ectopic gene conversion (GC; Lys+ colonies). Colonies isolated from plates with various concentrations of MMS were subjected to fluctuation tests, and the rates calculated as described (liefshitz *et al.* 1995). The MMS concentrations used were low and did not cause cell death in the wt strain.

### Western blot analysis

Cells were lysed with the B60 buffer (HEPES 50mM, Triton 100X 0.1%, β-glycerophosphate 20mM, Potassium Acetate 60mM, Glycerol 10%) with added protease inhibitors (Roche cat # 11836145001) and DTT (Millipore, cat # 578517). Mechanical lysis of the cells was performed by using glass beads (Sigma cat # G-92680). The level of Srs2 protein was assessed in mid-logarithmic cultures using an anti-Srs2 antibody (yC-18 Santa–Cruz) diluted to 1:750. Actin levels were used as loading controls (ab3280, Abcam).

### Doubling time measurements

Independent cultures of each genotype were grown to mid-logarithmic phase, diluted to ∼1 × 10^6^ cells/ml, MMS was added to the appropriate concertation and incubated in 96-well plates at 30°. OD_600_ was measured automatically every 30 min by a TECAN infinite M200 pro incubator and spectrophotometer. Generation time was calculated from the growth curve in the logarithmic growth period using the following formula: log_2_ (OD_600_, ΔOD), where ΔOD is [OD at time point X- OD at time point X+1]. The slope of the curve obtained by plotting these values against time was normalized to the growth rate of the wt strain as described (singh *et al.* 2013).

### Microscopic examination

Independent cultures of each genotype were grown to mid-logarithmic phase; MMS was added to the appropriate concertation and incubated for another 3 hr to allow the completion of a least one cell division. Cell cycle phase was determined by the cell morphology under microscope.

### RNA and qPCR

Total cellular RNA was isolated from different strains using MasterPure yeast RNA purification kit (Epicentre Biothechnologies). Reverse-transcription was carried out using qScript cDNA synthesis kit (Quanta biosciences), followed by quantitative PCR with primers specific for each ORF. RNA levels were determined relative to a control gene, ACT1. The following primers were used:

ACT1 F GAAAAGATCTGGCATCATACCTT
ACT1 R AAAACGGCTTGGATGGAAAC‘
RDH54 + 1794F AACTCTCCTGGATTGGTTGGCT
RDH54 + 1951R CGACCTTCTCCTTGGTACCCTT

### Data availability

All strains and plasmids are available upon request (described in Tables 1 and 2).

## Results

### Addition of an extra copy of SRS2 sensitizes the cells to MMS in haploid strains

**Figure 1:**
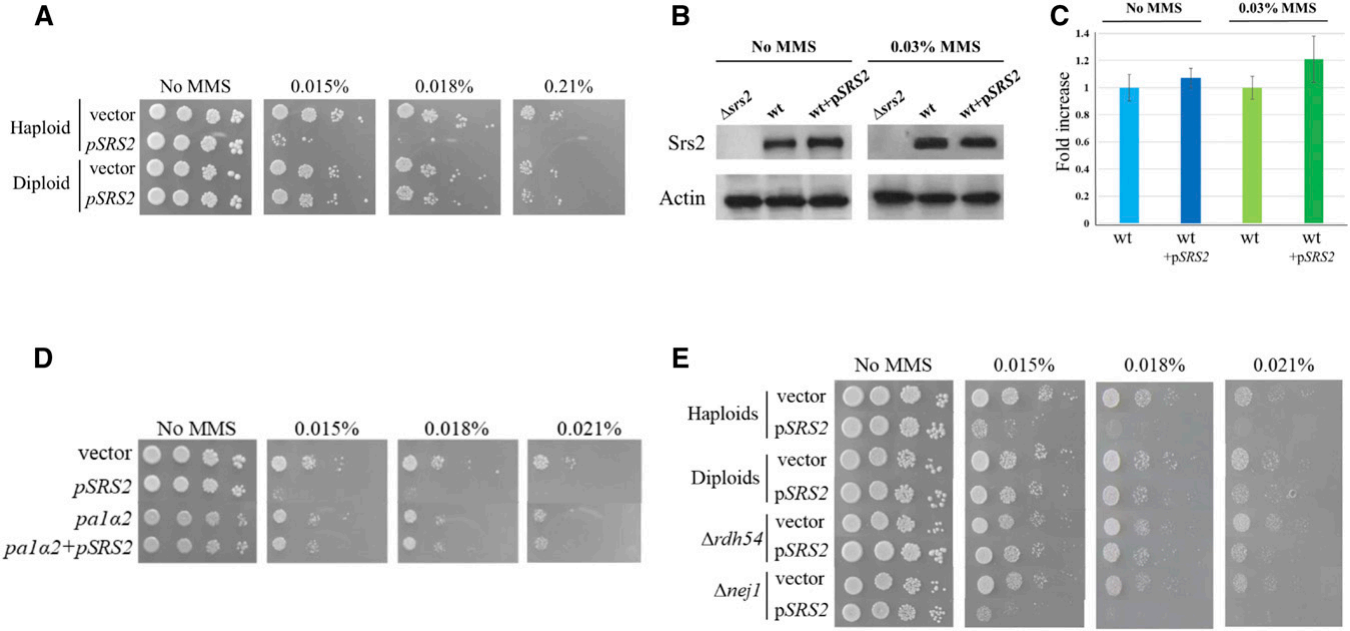
A single extra copy of *SRS2* sensitizes wt cells to MMS: (A) Haploids but not diploids show sensitivity to MMS when extra copy of *SRS2* on a centromeric plasmid is added. Ten-fold serial dilutions were plated on YEPD plates with increasing amounts of MMS. (B,C) Addition of the *SRS2*-carrying plasmid does not show a significant change in protein levels. (D) Mimicking diploid gene expression in haploids or deleting *RDH54*, but not *NEJ1* (E) abolishes the sensitization effect.

Srs2 plays an important role in the regulation of homologous recombination, as it is able to displace the strand exchange protein Rad51 from DNA. The regulation of Srs2 activity is not well-understood. Strong overexpression of this protein results in cell lethality when combined with mutations in genome stability maintenance genes (León Ortiz *et al.* 2011). We noticed that a single, extra copy of the *SRS2* gene on a centromeric plasmid causes sensitization of wt cells to DNA damaging agents, without affecting cell growth. Figure 1A shows that cells carrying two copies of *SRS2* (one genomic, another on a centromeric plasmid) were unable to form colonies on plates containing 0.015% MMS or higher concentrations. Western blot analysis showed that the extra *SRS2* copy only very slightly increased the level of Srs2 in the cells, in the absence of DNA damage (<10% increase) or even in the presence of MMS (∼20% increase under exposure to the highest MMS doses, Figure 1B,C, less than 10% under lower doses).

It was previously reported that a deletion of *SRS2* has a stronger phenotype in diploids than in haploids (aboussekhra *et al.* 1989; gazy *et al.* 2013). We therefore tested the effect of an extra copy of Srs2 in isogenic diploid cells, and found that expressing a single extra copy of Srs2 in diploids does not cause sensitization (Figure 1A). Protein levels of Srs2 in diploid strains, however, were not different from those of haploid strains, in the presence or absence of an extra copy of *SRS2* (Figure S1).

The *MAT* locus specifies mating type in yeast. Haploid cells carry either the *MAT****a*** or the *MAT****α*** allele. Once a zygote is formed by mating, the presence of both *MAT* alleles leads to the creation of the a1/α2 protein complex (encoded jointly by the *MAT****a*** and *MAT****α*** alleles), which represses the expression of haploid-specific genes, and allows the expression of diploid-specific genes [reviewed in (haber 2012)]. Expression of the a1/α2 complex in haploids causes a shift from error prone repair to recombinational repair (valencia *et al.* 2001; valencia-burton *et al.* 2006). Furthermore, it was shown that diploids deleted for *SRS2* are more sensitive if they express the a1/α2 complex, compared to diploids homozygous for the *MAT* locus. This was interpreted to mean that the a1/α2 complex promotes HR, and in the absence of an anti-HR activity by Srs2 cells try to repair lesions by HR at the G1 phase, where HR is inefficient (heude and fabre 1993). Therefore, diploids might be resistant to the sensitization effect due to the different gene expression controlled by the a1/α2 protein complex.

To mimic the gene expression of diploids in haploids we introduced a plasmid carrying the a1/α2-encoding genes into haploid wild type cells together with p*SRS2*. Figure 1D shows that expressing a1/α2 complex abolishes the sensitization effect of Srs2 in haploid cells. The a1/α2 suppression of sensitization prompted us to search for genes affected by this complex that may mediate the suppression effect. Previous bioinformatic efforts resulted in the identification of a number of genes regulated by the a1/α2 complex (Galgoczy *et al.* 2004; Nagaraj *et al.* 2004). Deleting several of these differentially expressed genes: *DDR2*, *FAR1*, *FUS3*, *GPA1* or *AMN1* did not prevent the sensitivity to MMS when extra Srs2 was added (data not shown). Only two genes in the differentially expressed genes list are directly involved in DNA damage response: *NEJ1* and *RDH54*. *NEJ1* is a cell-type specific regulator essential to non-homologous end joining (kegel *et al.* 2001; valencia *et al.* 2001; valencia-burton *et al.* 2006). Nej1 was shown to recruit Srs2 to DNA double-strand breaks, and to support repair by a single-strand annealing-like mechanism (carter *et al.* 2009). *RDH54* is a member of the *SNF2* family (klein 1997; anand *et al.* 2014; tsaponina and haber 2014), which possesses a dsDNA-dependent ATPase activity that can promote its translocation on dsDNA, resulting in DNA supercoiling and transient strand unwinding (eisen *et al.* 1995). Both Srs2 and Rdh54 physically interact with the recombinase Rad51 and synergize with the Rad51–ssDNA nucleoprotein filament to promote D-loop formation, DNA branch migration and chromatin remodeling, all of which are essential steps in HR (san filippo *et al.* 2008; anand *et al.* 2014). We tested the effect of Srs2 sensitization in the background of *Δnej1* and Δ*rdh54*. Deletion of *NEJ1* did not cause any changes in the sensitization phenotype. In contrast, deletion of *RDH54* abolished the sensitization caused by p*SRS2* (Figure 1E), indicating that Rdh54 activity is necessary for the toxicity observed in haploid cells. We confirmed that the transcription of *RDH54* is indeed downregulated in diploids by measuring its mRNA levels (Figure S1B). As expected, it is sufficient to express the a1/α2 complex in haploids to repress the expression of *RDH54*; reciprocally, deletion of one of the *MAT* alleles in diploids restores high *RDH54* expression (Figure S1B). We infer from these results that in the presence of DNA damage increased Srs2 levels interfere with the proper DNA repair process by a mechanism that also involves Rdh54 activity.

### An extra copy of Srs2 does not affect cell cycle progression

Next, we set to understand the nature of the MMS sensitization effect. Srs2 has a role in the activation of the Rad53-dependent DNA damage response checkpoint (liberi *et al.* 2000). In addition, Srs2 is involved in recovery and adaption from checkpoint-mediated cell cycle arrest (liberi *et al.* 2000; vaze *et al.* 2002). We hypothesized that an extra copy of Srs2 may over-activate the checkpoint, and prevent or interfere with normal cell cycle progression, similarly to what was shown with strong Srs2 overexpression in certain backgrounds (León Ortiz *et al.* 2011). First, we tested whether the addition of an extra copy of *SRS2* affects cell cycle progression or cell division. *SRS2* did not extend the cells’ doubling time; moreover, microscopic examination showed no differences in cell cycle distribution between cells carrying an extra copy of Srs2 or a control plasmid (Figure S2A). Mutation in genes involved in checkpoint activation, such as *RAD24*, which loads the 9-1-1 checkpoint clamp (majka and burgers 2003) or the checkpoint adaptors *RAD9* (weinert and hartwell 1988; toh and lowndes 2003) or Mrc1(alcasabas *et al.* 2001) had no effect either (Figure S2B). These results led us to the conclusion that the *SRS2* sensitivity cannot be attributed to an impairment of the DNA damage or replication checkpoint activation and progression.

### Unregulated levels of Srs2 cause inhibition of DNA repair

**Figure 2:**
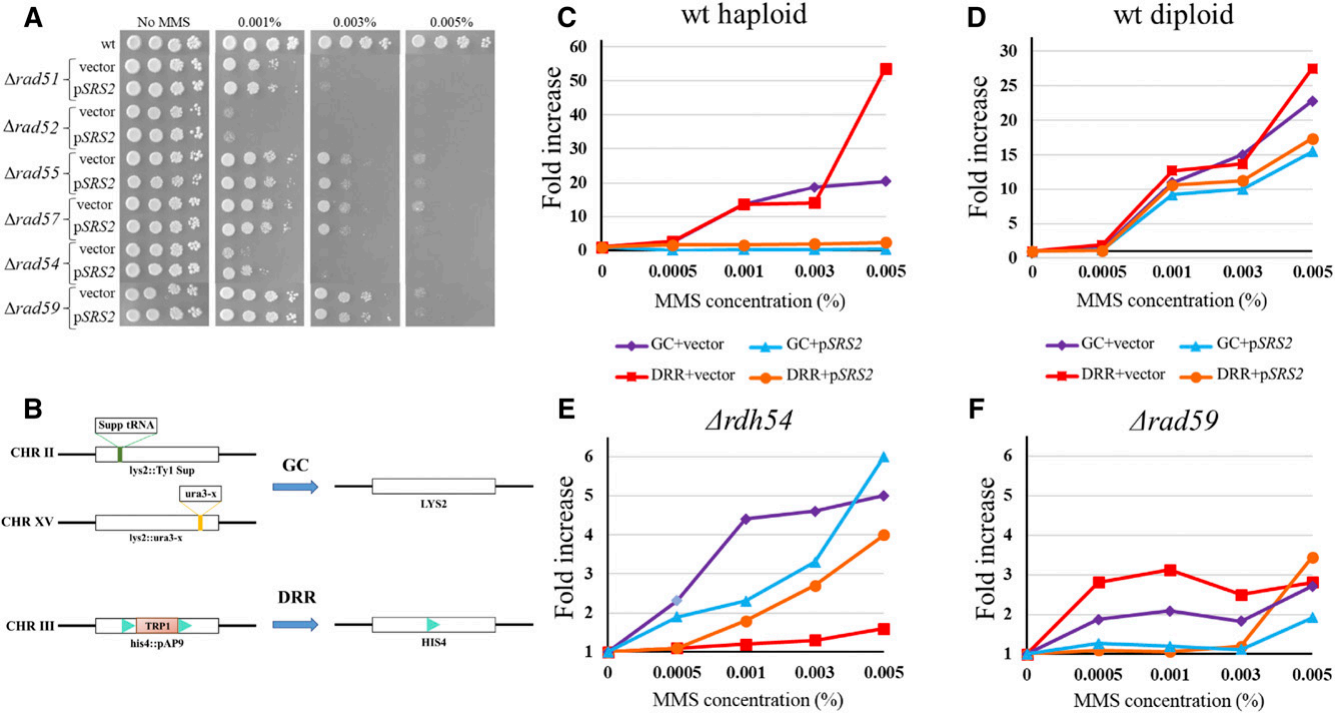
Analysis of the role of Srs2 on induced DNA repair: (A) The sensitization effect of *SRS2* requires the HR machinery. (B) A schematic representation of strain MK166 and its recombination substrates. (C) DNA damage induces recombination levels (gene conversion and direct-repeat recombination) in wt cells. An extra copy of *SRS2* reduces the HR levels. (D) The induced HR in diploid cells is not affected by an extra copy of *SRS2*. (E, F) in the background of *Δrdh54* or *Δrad59* there is less GC and DRR induction. An extra copy of *SRS2* does not further reduce this effect.

After we excluded checkpoint involvement, we turned to the better-characterized functions of Srs2, its involvement in homologous recombination. We tested whether deletion of the HR machinery genes can abolish the *SRS2* sensitization effect. Figure 2A shows that this was indeed the case with *RAD51*, *RAD52*, *RAD55*, and *RAD57*. Figure 2A also shows a very slight, but consistent *increase* in MMS resistance in *Δrad54* cells when p*SRS2* is added (see Discussion). We conclude that the MMS sensitivity observed upon addition of an extra copy of *SRS2* is due to its effect on the activity of the HR pathway.

To monitor the effect of extra Srs2 expression on the levels of recombination we used strain MK166. This strain carries several substrates to measure HR; here we monitored recombination between two direct repeats at the *HIS4* gene (direct repeat recombination, DRR), which results in His+ colonies, and non-reciprocal recombination (gene conversion, GC) between two disrupted copies of the *LYS2* gene, which results in the creation of Lys+ colonies. Whereas DRR is essentially Rad51-independent, GC depends completely on Rad51 filament formation (liefshitz *et al.* 1995) (Figure 2B). We measured recombination rates in strains carrying p*SRS2* or a control vector, in cells untreated or subjected to prolonged exposure to very low doses of MMS, which did not reduce viability.

Figure 2C and Figure S3A show that increasing amounts of MMS lead to a 20-fold increase of HR by gene conversion (GC) in haploid strains bearing an empty vector. In contrast, extra expression of *SRS2* prevented HR induction. Interestingly we noticed that not only the GC rates were reduced in the presence of an extra copy of *SRS2*. The rate of DRR, which is mostly Rad51-independent (liefshitz *et al.* 1995), was increased more than 50-fold by MMS exposure; the presence of an extra single copy of Srs2 completely abolished such an induction (Figure 2C, Figure S3B). This fact was surprising, and might suggest that the sensitization effect is caused not by the lack of removal of Rad51, but rather by some other function of Srs2. Strikingly, the effect of Srs2 excess on HR was seen only in haploid cells, and not in diploids (Figure 2D, Figure S3A and B), consistent with the lack of sensitization to MMS observed (Figure 1A).

Deletion of *RDH54* abolished the sensitization effect on MMS in haploids, similarly to what was seen in diploids (Figure 1E). We therefore tested the effect of deleting *RDH54* on the induction of HR. Previous studies revealed that *Δrdh54* has no defects in intrachromosomal GC or DDR during normal cell cycle progression (klein 1997). We confirmed these results for spontaneous levels of HR. However, the induction of HR was much lower than in wt: GC was 4 times lower and the induced DRR was almost completely abolished, suggesting that Rdh54 has a role in DNA damage induced SSA (Figure 2E, Figure S3C). However, in contrast to the wt strain, the addition of *SRS2* did not further reduce GC or DRR in the strain without *RDH54*; on the contrary, it even led to a small increase in DRR induction (Figure 2E, Figure S3D). These results again support a model in which, in the presence of DNA damage, Srs2 has a negative effect, which depends on Rdh54.

Since deletion of *RAD59*, which usually has a minor role in DNA repair, also abolished the sensitization effect (Figure 2A), we also tested its effects on HR. Similar to what was seen in *Δrdh54* strains, in *Δrad59* strains the levels of induced GC and DRR were reduced by 7.5- and 18-fold respectively and were unaffected by Srs2 overexpression (Figure 2F, Figure S3C and D). These results places Rad59 with Rdh54 in the pathway that inhibits repair when Srs2 is overexpressed. The similar results obtained in Rad51-dependent GC and Rad51-independent DRR suggest that the toxic activity of Srs2 is unrelated to its function in dismantling Rad51 filaments (krejci *et al.* 2003; veaute *et al.* 2003). Yet, as Figure 2A shows, an active HR pathway is required.

### Identifying SRS2 motifs and modifications important for its sensitization effect

To investigate which elements of the Srs2 protein play a role in the sensitization effect, we used a set of deletions and point mutations that affect various known functions of Srs2 or its regulation. All the mutants were present in single-copy centromeric plasmids; a wt Srs2 gene and an empty vector served as appropriate controls.

**Figure 3:**
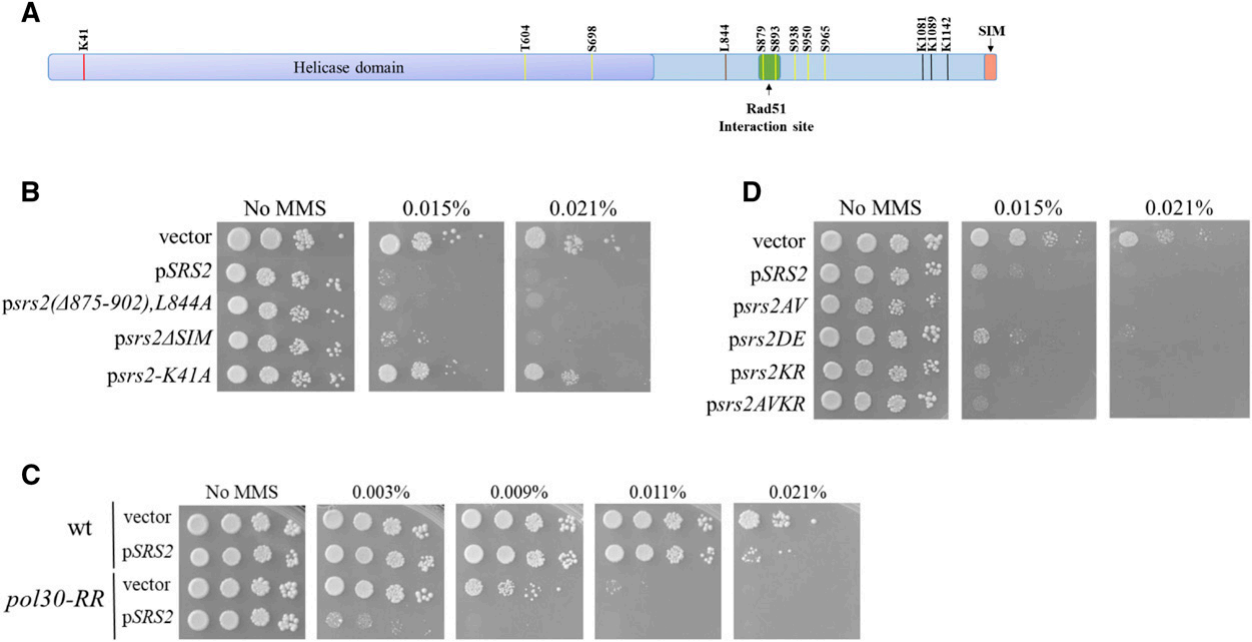
Analysis the role of Srs2 on induced DNA repair: (A) the sensitization effect of *SRS2* requires the HR machinery. (B) A schematic representation of strain MK166, which allows scoring of recombination levels for different substrates. (C) An extra copy of *SRS2* reduces the induced levels of GC and DRR in haploids. (D) Diploids are not affected by an extra copy of *SRS2*. (E)(F) In the background of *Δrdh54* or *Δrad59* there is less GC and DRR induction, however an extra copy of *SRS2* does not show any negative effect on HR repair.

The helicase domain of Srs2 occupies about two thirds of the protein (Figure 3A). ATP hydrolysis was showed to be needed both for the helicase and the Rad51 dismantling functions of *SRS2* (krejci *et al.* 2004). The *srs2-K41A* mutant is unable to hydrolyze ATP, and renders Srs2 completely inactive. Accordingly, whereas the wt *SRS2* gene on a centromeric plasmid confers sensitivity, the *srs2-K41A* allele fails to do so (Figure 3B).

Downstream to the helicase domain is a region (aa 875-902) that was defined as critical for the interaction with Rad51 (colavito *et al.* 2009). A point mutation (L844A) was also characterized, which abolishes the Rad51-Srs2 interaction (islam *et al.* 2012). We thus created a double mutant [*srs2(Δ875-902),L844A*] to make completely sure that the interaction with Rad51 is abolished. Remarkably, addition of an Srs2 protein that completely lacks Rad51 interaction motifs still caused the same level of MMS sensitivity as the addition of wt Srs2 (Figure 3B). This result confirms that despite the requirement for Rad51 and other HR proteins (Figure 2A), the MMS sensitivity caused by an addition of an extra copy of *SRS2* is independent of Srs2 interaction with Rad51.

We also tested the *srs2ΔSIM* mutant, which lacks the last 6 amino acids that are important for its interaction with SUMO, and play a role in recruiting Srs2 to SUMOylated PCNA (pfander *et al.* 2005). Srs2 without the SIM motif still exhibited the sensitization effect (Figure 3B) indicating that the interaction with SUMOylated PCNA is not required. The finding that the sensitization phenotype does not require the binding of *SRS2* to SUMOylated PCNA was surprising because Srs2 plays a role in HR-mediated repair during DNA replication (pfander *et al.* 2005) and PCNA interaction is necessary for the synthetic sickness of DNA replication and repair mutants in the presence of high levels of Srs2 protein (León Ortiz *et al.* 2011). To validate that Srs2 does not need to bind SUMOylated PCNA, we checked the effect of an additional copy of wt *SRS2* in a *pol30-RR* strain, which is unable to undergo modifications on PCNA (hoege *et al.* 2002). Consistent with our previous findings, Figure 3C shows that in a *pol30-RR* background an extra copy of Srs2 still causes MMS sensitivity.

After examining the requirement of the known motifs in Srs2, we tested the importance of specific protein modifications to the sensitization effect. Srs2 contains 7 mapped phosphorylation sites and 3 SUMOylation sites (Figure 3A). We tested 4 different *srs2* alleles 1) p*srs2AV* expresses an Srs2 protein that cannot be phosphorylated (2) In p*srs2DE* the mutations mimic constant phosphorylation 3) An Srs2 mutant that cannot be SUMOlyted (p*srs2KR*) and 4) A combined allele of Srs2 that is neither phosphorylated nor SUMOlyted (p*srs2AVKR*) (saponaro *et al.* 2010). Extra expression of all these Srs2 mutants still showed a sensitization effect (Figure 3D).

In summary, our results imply that cells become sensitive to MMS induced DNA damage in the presence of small increases in the level of the Srs2 protein by a process that requires its helicase activity, but does not require an interaction with Rad51 or post-translational modification of PCNA or of Srs2 itself.

### The Srs2 helicase activity is responsible for processing DNA Into toxic intermediates

Srs2 was previously proposed to promote the generation of toxic intermediates when the Rad6/Rad18-dependent DNA damage tolerance (DDT) pathway is not available. The sensitivity to DNA damaging agents of mutants in this pathway (*Δrad5,Δrad18*, *pol30-K164R*) is therefore suppressed by deleting *SRS2* (lawrence and christensen 1979; aboussekhra *et al.* 1989; papouli *et al.* 2005; pfander *et al.* 2005). Furthermore, this suppression effect is abolished when *RAD51* is also removed (schiestl *et al.* 1990; pfander *et al.* 2005). From these experiments, it was deduced that the cause of the toxicity is the constant removal of Rad51 by Srs2, which prevents the cells from taking advantage of the HR repair pathway. Once *SRS2* is deleted, it can no longer remove Rad51 and thus the more efficient recombinational repair can be utilized. Accordingly, if *RAD51* is deleted in these strains, then the repair is not channeled through Rad51 and hence the absence of Srs2 has no effect. Our results, in contrast, suggest a different explanation for the sensitization caused by extra Srs2: an extra copy of Srs2 impairs proper DNA repair through its helicase domain but not by its ability to dislodge Rad51.

**Figure 4:**
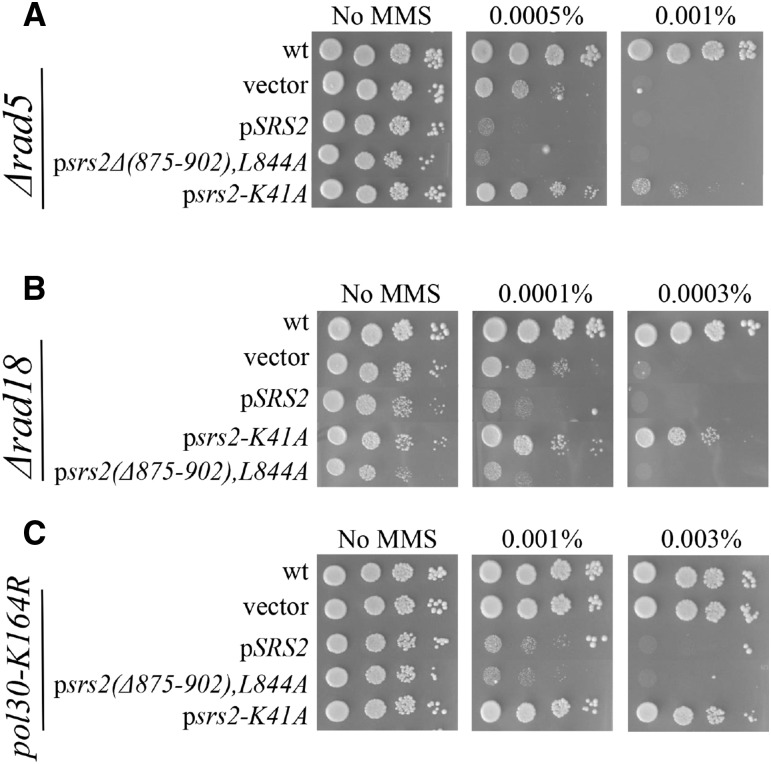
The sensitization effect of Srs2 is independent of the DDT pathway: Cells were transformed with centromeric plasmids and plated on YEPD plates with various MMS concentrations. Ten-fold serial dilutions are shown. (A) a *Δrad5* strain (B) a *Δrad18* strain (C) a *pol30-K164R* strain.

To further characterize this effect, we first tested whether the sensitization effect is still present when the Rad6/Rad18 pathway is impaired. We introduced wt Srs2, *srs2-K41A* (no ATPase activity) and the *srs2(Δ875-902),L844A* (no Rad51 interaction) allele in strains with impaired DDT pathway (*Δrad5*, *Δrad18*, *pol30-K164R*). Figure 4 shows that the *SRS2* sensitization effect is independent of the DDT pathway, as it is clearly detected in DDT mutants carrying an extra copy of wt *SRS2* or *srs2(Δ875-902),L844A* allele. As with previous results, the helicase-dead allele did not show any sensitization and even made the strains slightly more resistant to MMS, indicating that it may counteract the inhibitory effect of native Srs2 when the Rad6/Rad18 pathway is inactive.

**Figure 5:**
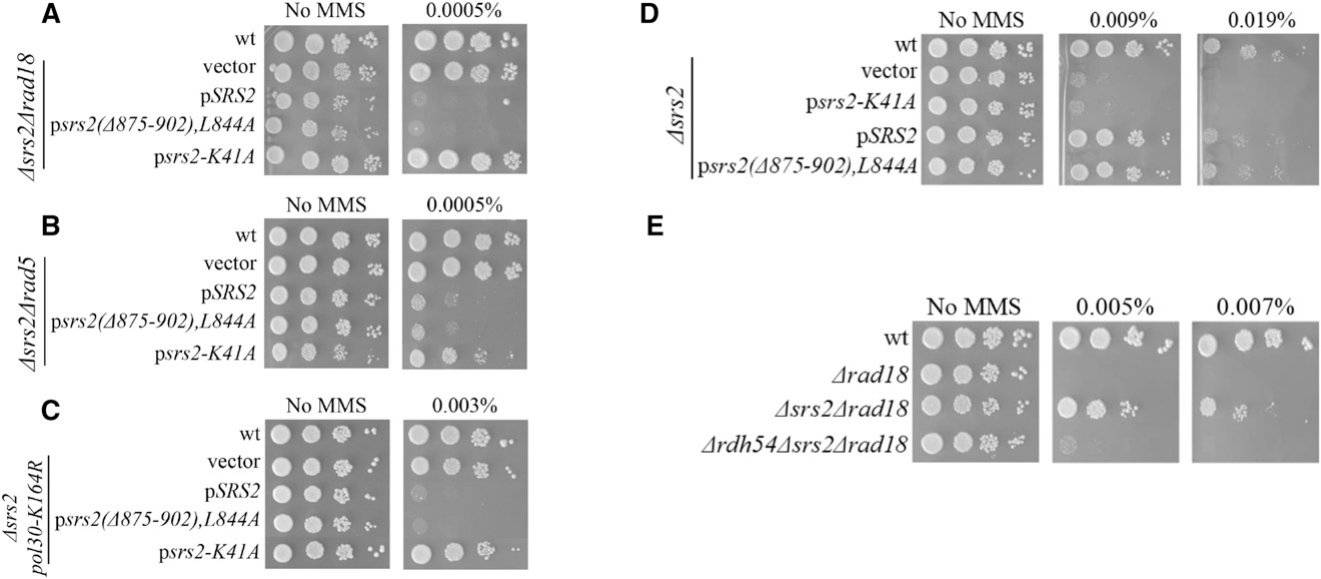
The DNA helicase activity of Srs2 is responsible for the creation of toxic intermediates: Cells were transformed with centromeric plasmids and plated on YEPD plates with various MMS concentrations. Ten-fold serial dilutions are shown. (A) a *Δsrs2 Δrad18* strain (B) a *Δsrs2 Δrad5* strain (C) a *Δsrs2 pol30-K164R* strain. (D) Interaction with Rad51 is not necessary for Srs2 to cope with DNA damage caused by MMS. (E) Deletion of *RDH54* suppresses the creation of toxic intermediates by *SRS2*, in a DDT deficient strain.

Next, we tested what mutations can still suppress the sensitivity to MMS of DDT- impaired cells. We introduced the mutants on a plasmid in cells deleted for *SRS2* and defective in the Rad6/Rad18 repair pathway. When wt *SRS2* was expressed in *∆srs2 Δrad18*, *Δsrs2 Δrad5* or *Δsrs2 pol30-K164R* strains, it sensitized the cells to MMS, as expected (Figure 5). In contrast, and also as expected, the helicase-dead *srs2-K41A* failed to complement the *Δsrs2* mutation and showed no sensitivity in the absence of an active DDT pathway. Importantly, when the mutant that cannot interact with Rad51was tested, it also restored MMS sensitivity, comparable to that conferred by the wt Srs2 protein. The *srs2(Δ875-902),L844A* allele was also able of fully complementing a strain deleted for the *SRS2* gene (Figure 5D), further supporting the observation that Srs2 does not require dismantling Rad51 nucleofilament to deal with DNA damage caused by MMS. These results show that the Srs2 toxic intermediates are not caused by Rad51 removal, but are rather due to Srs2’s helicase function. Consistent with the proposed role of Rdh54 in the sensitization mechanism, a deletion of *RDH54* restores sensitivity to MMS to a *Δsrs2 Δrad18* mutant (Figure 5E).

## Discussion

### The DNA helicase function of Srs2 and not its activity in evicting Rad51 is required to deal with DNA damage and cause the creation of toxic intermediates

In this work, we show that even a very slight over expression of Srs2 causes MMS sensitivity. We were unable to detect significant differences in the protein levels by Western blot analysis. This implies that even extremely small differences in protein levels have a biological significance. This effect is eliminated upon deletion of the HR pathway genes: Rad51, Rad52, Rad54, Rad55 and Rad57, which suggests that excess Srs2 only causes the creation of toxic intermediates after the initiation of homologous recombination repair. Interestingly our fluctuation analysis show that not only gene conversion but also the direct-repeat recombination pathway, which works mainly by a Rad51-independent mechanism (single-strand annealing), is inhibited when an extra copy of Srs2 is introduced. These results imply that the inhibition of DNA repair is not dependent on the removal of Rad51 by Srs2 *per se*. Indeed, when an extra copy of Srs2 which is unable to bind Rad51 [*srs2 (Δ875-902),L844A*] (colavito *et al.* 2009; islam *et al.* 2012) was introduced, it had the same sensitization effect as wt Srs2 (Figure 3B). However, the helicase dead mutant (*srs2-K41A)* had no effect, showing that the lack of efficient repair caused by the Srs2’s helicase activity on DNA metabolism is responsible for the observed phenotypes. As further proof for a helicase-dependent and Rad51-removal-independent activity of Srs2, we introduced Srs2 plasmids carrying these mutations into strains impaired in the DNA damage tolerance pathway. In these genetic backgrounds (*Δrad18*, *Δrad5* or *pol30-K164R*), Srs2 creates toxic intermediates that sensitize cells to DNA damaging agents (Figure 4). Deletion of *SRS2* suppresses this sensitivity; whereas the wt and the Rad51-interaction-defective *SRS2* alleles could complement the *Δsrs2* phenotype, restoring sensitivity, the helicase dead mutant was unable to do so (Figure 5A,B,C). These results confirm that the toxic intermediates are caused by Srs2’s helicase activity and not by Rad51 eviction. We show, however, that the toxic effect requires an attempt by the cells to repair the damage by HR.

### SRS2 toxic intermediates created by slight overexpression are independent of SUMOlyed PCNA and checkpoint activation

Leon Ortiz and co-workers (León Ortiz *et al.* 2011) carried out a screen for mutants that show synthetic dosage lethality with strong overexpression of Srs2. They identified a relatively large number of mutants, affecting selected cellular functions, including DNA and RNA metabolism, mitochondrial and ribosomal functions and vesicular traffic. The sensitivity to DNA damaging agents that we observe upon a very slight increase in Srs2 levels (Figures 1B and Figure S1A) in wild type cells seems to be due to a different mechanism of action, specific for DNA damage situations: First, the sensitization phenotype is only visible in the presence of DNA damage. Second, whereas strong Srs2 overexpression elicits and requires the DNA damage checkpoint (León Ortiz *et al.* 2011), we did not see any similar effect (data not shown), nor did we see changes in cell cycle distribution. Moreover, deletion key proteins of the checkpoint activation genes (*RAD9*, *MRC1* or *RAD24)*, did not have any effect on the sensitization by Srs2 increased levels (Figure S1B). Third, whereas the synthetic dose lethality observed by Leon Ortiz *et al.* was independent of Srs2’s helicase activity, the sensitization we observe is lost if the helicase is inactivated by mutation (Figure 3B). Furthermore, unlike the results obtained with strong overexpression, a single extra copy does not require an interaction with SUMOlated PCNA (Figure 3C). Thus, our results point to an additional role for Srs2, which impedes proper DNA repair when its levels are even slightly increased.

### The role of Srs2 phosphorylation and SUMOylation

To further investigate how unregulated Srs2 might affect DNA repair, we tested Srs2 mutants deficient in phosphorylation and SUMOylation. Srs2 phosphorylation is carried out by Cdk1 (Cdc28) (liberi *et al.* 2000; ubersax *et al.* 2003; deshmukh *et al.* 2016), and it has been proposed that this is needed to dismantle specific DNA structures (saponaro *et al.* 2010), such as the D- loops, in a helicase-dependent manner during homologous recombination repair. Phosphorylation of Srs2 is also required to complete the SDSA pathway (saponaro *et al.* 2010), and thus for HR-dependent recovery following chronic DNA damage exposure (hishida *et al.* 2010). The role of Srs2 SUMOylation is less understood. Mutation of the 3 target lysines can suppress the defect of Srs2 alleles which cannot undergo phosphorylation, suggesting that SUMOylation of Srs2 might have some inhibitory effect on the SDSA repair pathway (saponaro *et al.* 2010). In addition, it was shown that interaction between SUMOylated PCNA and Srs2 inhibits Srs2 SUMOylation (kolesar *et al.* 2012). Our results (Figure 3) show that none of these modifications has an effect on Srs2-dependent sensitization. Thus, even slight increases in Srs2 levels seem to completely overrule the complex regulatory mechanisms that keep its activity in check.

### Haploid *vs.* diploid regulation

Our results suggest that haploids and diploids employ different strategies to cope with lesions in their DNA. Whereas GC was induced by MMS to higher levels in diploids, (in comparison to isogenic haploids), the opposite seems to be the case for DRR. This probably reflects the fact that GC (by SDSA) in haploid cells, which usually involves the sister chromatid, is restricted to the relatively short period after a chromosome has duplicated, and before the two sisters separate at anaphase(Machín *et al.* 2016; Lin and O’Connell 2017). In contrast, diploid cells have an additional source of donors for gene conversion throughout the cell cycle in the homologous chromosome. In haploids, uncontrolled activity of Srs2 inhibits both GC and DRR, leading to cell death; in contrast, diploids escape this fate by using the alternative homology source as partner for repair by SDSA, and by attenuating the DDR repair by reducing gene expression of Rdh54 (Durdiková and Chovanec 2017).

*Δrdh54* haploid strains showed spontaneous GC levels comparable to those of the wt, but strongly reduced the induction of HR when cells were exposed to MMS (Figure 2D). Our results suggest that Rdh54 may not play a major role during normal DNA replication, but it could be activated when cells are exposed to genotoxins and have to deal with more severe DNA damage. Diploid cells, which repress *RDH54* expression, may therefore rely more on Srs2 activity. Consistently, previous work has shown that *Δsrs2* diploids are more sensitive to DNA damaging agents than isogenic haploids (aboussekhra *et al.* 1989; gazy *et al.* 2013), although its protein levels are not increased (Figure S1A). Despite its reduced abundance, (Figure S1B) Rdh54 does play a role in diploids, as *Δrdh54/ Δrdh54* diploids exhibit reduced interchromosomal recombination levels (klein 1997). Moreover, *Δrdh54* and *Δsrs2* show a synthetic sickness in diploids, consistent with overlapping roles between the helicases, even in diploids.

### Srs2 DNA helicase possible mechanisms of action

During normal replication, cells deal with spontaneous damage by a variety of repair mechanisms. In yeast, most of this damage is silently repaired by HR with a sister chromatid (fabre *et al.* 1984; kadyk and hartwell 1992). During normal cell cycle progression, the error-free DDT pathway deals with most of the spontaneous DNA damage. However, when there is extensive damage, as in cells exposed to genotoxic drugs, cells activate repair by HR in a genome-wide fashion. By interfering with the productivity of this mechanism through slight overexpression of Srs2, we have uncovered a requirement for Rdh54 and Rad59 in this induced pathway. Strains lacking these proteins show wt levels of spontaneous HR, but reduced induction of HR, which is not affected by Srs2 overexpression (Figure 2). Extra levels of Srs2 in wild type cells affect both GC and DRR, implying that the effect is due to a repair function of Srs2 that is unrelated to the removal of Rad51 (Figure 2C). Consistently, the toxic activity is seen even in the *srs2 (Δ875-902),L844A* allele, which is unable to bind Rad51 (colavito *et al.* 2009; islam *et al.* 2012).

What is the repair process affected by Srs2 overexpression? We know that it is a pathway that includes the HR proteins (Figure 2A), and also Rad59 and Rdh54. Rad59 plays a role in HR events that require annealing of complementary strands (davis and symington 2001; wu *et al.* 2006) and acts independently of Rad51(sugawara *et al.* 2000) (jablonovich *et al.* 1999). As Srs2, Rdh54 translocates along ssDNA, creating strand unwinding that may help in D-loop formation and branch migration (san filippo *et al.* 2008; anand *et al.* 2014). Interestingly we observed (Figure 2A) that *Δrad54* strains expressing an extra copy of Srs2 showed slightly less MMS sensitivity than those expressing an empty vector. Rad54 plays a role in HR at a later stage than Rad51, Rad52, Rad55 or Rad57 (wright and heyer 2014). Sister chromatid recombination is more dependent on Rad54 than on Rdh54 (arbel *et al.* 1999). In the absence of Rad54, increased Srs2 levels could discourage this repair substrate, encouraging alternative types of repair that may confer a slight resistance to MMS.

**Figure 6:**
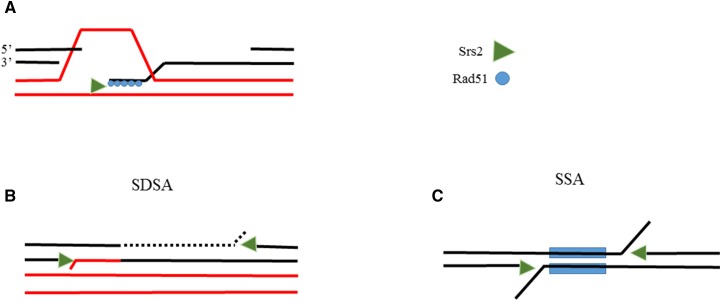
A model for Srs2 toxicity. (A) Srs2 regulates HR repair by removing Rad51 after invasion of a homologous sequence. This is necessary to allow the annealing of the newly synthesized DNA to the other broken arm. (B)(C) Srs2 activity can inhibit the completion of repair in both SDSA and SSA. This activity of Srs2 does not require interaction with PCNA or with Rad51, and depends only on Srs2’s helicase activity.

The requirement for Rad59 and Rdh54 activity suggests that unregulated levels of Srs2 might interfere with the last stages of HR, which involve annealing of complementary ssDNA and trimming of excess overhanging DNA flaps to allow ligation. Thus, following DNA damage, Srs2 participates in HR by removing Rad51 from the DNA (Figure 6A). This is necessary to allow disengagement of the invading strand and re-annealing to the broken arm. We propose that Srs2 can also act later to disrupt the annealed strands and inhibit the completion of the repair (Figure 6B). The helicase activity can unwind nicked, annealed DNA duplexes such as those created by the activity of Rad52 and Rad59 (Mortensen *et al.* 1996; Shinohara *et al.* 1998; Davis and Symington 2001). Similar configurations are created during SSA (Figure 6C). This later activity during HR repair, which is normally tightly regulated, increases upon overexpression of Srs2 and causes sensitivity to DNA damaging agents. The toxic activity is independent of contacts between Srs2 and PCNA, or of interactions with Rad51, and it only requires Srs2’s helicase activity.

### Concluding remarks

By studying the toxic effect of a slight overexpression of Srs2 in the presence a genotoxin, we have uncovered a mechanism that deals with DNA damage in haploid cells. Our results underscore the exquisite regulation needed to allow cells to cope efficiently with DNA damage without interfering with the repair itself, and uncover Rad51-independent roles of the Srs2 helicase.

## Supplementary Material

Supplemental Material is available online at www.g3journal.org/lookup/suppl/doi:10.1534/g3.118.200181/-/DC1.

## References

[bib1] Aboussekhra A. Chanet R. Zgaga Z. Cassier-Chauvat C. Heude M., 1989 RADH, a gene of Saccharomyces cerevisiae encoding a putative DNA helicase involved in DNA repair. Characteristics of radH mutants and sequence of the gene. Nucleic Acids Res. 17(18): 7211–7219. 10.1093/nar/17.18.7211 2552405 PMC334801

[bib2] Adamczyk J. Deregowska A. Panek A. Golec E. Lewinska A., 2016 Affected chromosome homeostasis and genomic instability of clonal yeast cultures. Curr. Genet. 62(2): 405–418. 10.1007/s00294-015-0537-3 26581629 PMC4826422

[bib3] Alcasabas A. A. Osborn A. J. Bachant J. Hu F. Werler P. J., 2001 Mrc1 transduces signals of DNA replication stress to activate Rad53. Nat. Cell Biol. 3(11): 958–965. 10.1038/ncb1101-958 11715016

[bib4] Anand R. P. Tsaponina O. Greenwell P. W. Lee C. S. Du W., 2014 Chromosome rearrangements via template switching between diverged repeated sequences. Genes Dev. 28(21): 2394–2406. 10.1101/gad.250258.114 25367035 PMC4215184

[bib5] Antony E. Tomko E. J. Xiao Q. Krejci L. Lohman T. M., 2009 Srs2 disassembles Rad51 filaments by a protein-protein interaction triggering ATP turnover and dissociation of Rad51 from DNA. Mol. Cell 35(1): 105–115. 10.1016/j.molcel.2009.05.026 19595720 PMC2711036

[bib6] Arbel A. Zenvirth D. Simchen G., 1999 Sister chromatid-based DNA repair is mediated by RAD54, not by DMC1 or TID1. EMBO J. 18(9): 2648–2658. 10.1093/emboj/18.9.2648 10228176 PMC1171344

[bib7] Aylon Y. Liefshitz B. Bitan-Banin G. Kupiec M., 2003 Molecular dissection of mitotic recombination in the yeast Saccharomyces cerevisiae. Mol. Cell. Biol. 23(4): 1403–1417. 10.1128/MCB.23.4.1403-1417.2003 12556499 PMC141147

[bib8] Bailly V. Lamb J. Sung P. Prakash S. Prakash L., 1994 Specific complex formation between yeast RAD6 and RAD18 proteins: a potential mechanism for targeting RAD6 ubiquitin-conjugating activity to DNA damage sites. Genes Dev. 8(7): 811–820. 10.1101/gad.8.7.811 7926769

[bib9] Boiteux S. Jinks-Robertson S., 2013 DNA repair mechanisms and the bypass of DNA damage in Saccharomyces cerevisiae. Genetics 193(4): 1025–1064. 10.1534/genetics.112.145219 23547164 PMC3606085

[bib10] Carter S. D. Vigasova D. Chen J. Chovanec M. Astrom S. U., 2009 Nej1 recruits the Srs2 helicase to DNA double-strand breaks and supports repair by a single-strand annealing-like mechanism. Proc. Natl. Acad. Sci. USA 106(29): 12037–12042. 10.1073/pnas.0903869106 19571008 PMC2715516

[bib11] Colavito S. Macris-Kiss M. Seong C. Gleeson O. Greene E. C., 2009 Functional significance of the Rad51-Srs2 complex in Rad51 presynaptic filament disruption. Nucleic Acids Res. 37(20): 6754–6764. 10.1093/nar/gkp748 19745052 PMC2777448

[bib12] Davis A. P. Symington L. S., 2001 The yeast recombinational repair protein Rad59 interacts with Rad52 and stimulates single-strand annealing. Genetics 159: 515–525. 11606529 10.1093/genetics/159.2.515 PMC1461847

[bib13] Deshmukh A. S. Agarwal M. Dhar S. K., 2016 Regulation of DNA replication proteins in parasitic protozoans: possible role of CDK-like kinases. Curr. Genet. 62(3): 481–486. 10.1007/s00294-015-0562-2 26780367

[bib14] Dmowski M. Fijalkowska I. J., 2017 Diverse roles of Dpb2, the non-catalytic subunit of DNA polymerase epsilon. Curr. Genet. 63(6): 983–987. 10.1007/s00294-017-0706-7 28516230 PMC5668336

[bib15] Durdiková K. Chovanec M., 2017 Regulation of non-homologous end joining via post-translational modifications of components of the ligation step. Curr. Genet. 63(4): 591–605. 10.1007/s00294-016-0670-7 27915381

[bib16] Eisen J. A. Sweder K. S. Hanawalt P. C., 1995 Evolution of the SNF2 family of proteins: subfamilies with distinct sequences and functions. Nucleic Acids Res. 23(14): 2715–2723. 10.1093/nar/23.14.2715 7651832 PMC307096

[bib17] Fabre F. Boulet A. Roman H., 1984 Gene conversion at different points in the mitotic cycle of Saccharomyces cerevisiae. Mol. Gen. Genet. 195(1-2): 139–143. 10.1007/BF00332736 6387388

[bib18] Friedl A. A. Liefshitz B. Steinlauf R. Kupiec M., 2001 Deletion of the SRS2 gene suppresses elevated recombination and DNA damage sensitivity in rad5 and rad18 mutants of Saccharomyces cerevisiae. Mutat. Res. 486(2): 137–146. 10.1016/S0921-8777(01)00086-6 11425518

[bib19] Gadaleta M. C. Gonzalez-Medina A. Noguchi E., 2016 Timeless protection of telomeres. Curr. Genet. 62(4): 725–730. 10.1007/s00294-016-0599-x 27068713 PMC5056121

[bib20] Gaines W. A. Godin S. K. Kabbinavar F. F. Rao T. VanDemark A. P., 2015 Promotion of presynaptic filament assembly by the ensemble of S. cerevisiae Rad51 paralogues with Rad52. Nat. Commun. 6(1): 7834 10.1038/ncomms8834 26215801 PMC4525180

[bib80] Galgoczy D. J. Cassidy-Stone A. Llinás M. O'Rourke S. M. Herskowitz I., 2004 Genomic dissection of the cell-type-specification circuit in Saccharomyces cerevisiae. Proc. Natl. Acad. Sci. USA 2004 Dec 28 (52):18069-74. Epub 2004 Dec 16. 10.1073/pnas.0407611102 PMC535907 15604142

[bib21] Gangavarapu V. Prakash S. Prakash L., 2007 Requirement of RAD52 group genes for postreplication repair of UV-damaged DNA in Saccharomyces cerevisiae. Mol. Cell. Biol. 27(21): 7758–7764. 10.1128/MCB.01331-07 17785441 PMC2169055

[bib22] Gangloff S. Soustelle C. Fabre F., 2000 Homologous recombination is responsible for cell death in the absence of the Sgs1 and Srs2 helicases. Nat. Genet. 25(2): 192–194. 10.1038/76055 10835635

[bib23] Gao J. Kan F. Wagnon J. L. Storey A. J. Protacio R. U., 2014 Rapid, efficient and precise allele replacement in the fission yeast Schizosaccharomyces pombe. Curr. Genet. 60(2): 109–119. 10.1007/s00294-013-0406-x 24026504 PMC3954454

[bib24] Gazy I. Liefshitz B. Bronstein A. Parnas O. Atias N., 2013 A genetic screen for high copy number suppressors of the synthetic lethality between elg1Delta and srs2Delta in yeast. G3 (Bethesda) 3(5): 917–926. 10.1534/g3.113.005561 23704284 PMC3656737

[bib25] Haber J. E., 2012 Mating-type genes and MAT switching in Saccharomyces cerevisiae. Genetics 191(1): 33–64. 10.1534/genetics.111.134577 22555442 PMC3338269

[bib26] Heude M. Fabre F., 1993 a/alpha-control of DNA repair in the yeast Saccharomyces cerevisiae: genetic and physiological aspects. Genetics 133: 489–498. 8454201 10.1093/genetics/133.3.489 PMC1205337

[bib27] Hishida T. Hirade Y. Haruta N. Kubota Y. Iwasaki H., 2010 Srs2 plays a critical role in reversible G2 arrest upon chronic and low doses of UV irradiation via two distinct homologous recombination-dependent mechanisms in postreplication repair-deficient cells. Mol. Cell. Biol. 30(20): 4840–4850. 10.1128/MCB.00453-10 20713444 PMC2950541

[bib28] Hoege C. Pfander B. Moldovan G. L. Pyrowolakis G. Jentsch S., 2002 RAD6-dependent DNA repair is linked to modification of PCNA by ubiquitin and SUMO. Nature 419(6903): 135–141. 10.1038/nature00991 12226657

[bib29] Hofmann R. M. Pickart C. M., 1999 Noncanonical MMS2-encoded ubiquitin-conjugating enzyme functions in assembly of novel polyubiquitin chains for DNA repair. Cell 96(5): 645–653. 10.1016/S0092-8674(00)80575-9 10089880

[bib30] Ira G. Malkova A. Liberi G. Foiani M. Haber J. E., 2003 Srs2 and Sgs1-Top3 suppress crossovers during double-strand break repair in yeast. Cell 115(4): 401–411. 10.1016/S0092-8674(03)00886-9 14622595 PMC4493758

[bib31] Islam M. N. Paquet N. Fox D. 3rd Dray E. Zheng X. F., 2012 A variant of the breast cancer type 2 susceptibility protein (BRC) repeat is essential for the RECQL5 helicase to interact with RAD51 recombinase for genome stabilization. J. Biol. Chem. 287(28): 23808–23818. 10.1074/jbc.M112.375014 22645136 PMC3390654

[bib32] Jablonovich Z. Liefshitz B. Steinlauf R. Kupiec M., 1999 Characterization of the role played by the RAD59 gene of Saccharomyces cerevisiae in ectopic recombination. Curr. Genet. 36(1-2): 13–20. 10.1007/s002940050467 10447590

[bib33] Kadyk L. C. Hartwell L. H., 1992 Sister chromatids are preferred over homologs as substrates for recombinational repair in Saccharomyces cerevisiae. Genetics 132: 387–402. 1427035 10.1093/genetics/132.2.387 PMC1205144

[bib34] Kegel A. Sjostrand J. O. Astrom S. U., 2001 Nej1p, a cell type-specific regulator of nonhomologous end joining in yeast. Curr. Biol. 11(20): 1611–1617. 10.1016/S0960-9822(01)00488-2 11676923

[bib35] Klein H. L., 1997 RDH54, a RAD54 homologue in Saccharomyces cerevisiae, is required for mitotic diploid-specific recombination and repair and for meiosis. Genetics 147: 1533–1543. 9409819 10.1093/genetics/147.4.1533 PMC1208329

[bib36] Kolesar P. Sarangi P. Altmannova V. Zhao X. Krejci L., 2012 Dual roles of the SUMO-interacting motif in the regulation of Srs2 sumoylation. Nucleic Acids Res. 40(16): 7831–7843. 10.1093/nar/gks484 22705796 PMC3439891

[bib37] Kolodner R. D. Putnam C. D. Myung K., 2002 Maintenance of genome stability in Saccharomyces cerevisiae. Science 297(5581): 552–557. 10.1126/science.1075277 12142524

[bib38] Krejci L. Macris M. Li Y. Van Komen S. Villemain J., 2004 Role of ATP hydrolysis in the antirecombinase function of Saccharomyces cerevisiae Srs2 protein. J. Biol. Chem. 279(22): 23193–23199. 10.1074/jbc.M402586200 15047689

[bib39] Krejci L. Van Komen S. Li Y. Villemain J. Reddy M. S., 2003 DNA helicase Srs2 disrupts the Rad51 presynaptic filament. Nature 423(6937): 305–309. 10.1038/nature01577 12748644

[bib40] Lawrence C. W. Christensen R. B., 1979 Metabolic suppressors of trimethoprim and ultraviolet light sensitivities of Saccharomyces cerevisiae rad6 mutants. J. Bacteriol. 139: 866–876. 383698 10.1128/jb.139.3.866-876.1979 PMC218033

[bib41] León Ortiz A. M. Reid R. J. Dittmar J. C. Rothstein R. Nicolas A., 2011 Srs2 overexpression reveals a helicase-independent role at replication forks that requires diverse cell functions. DNA Repair (Amst.) 10(5): 506–517. 10.1016/j.dnarep.2011.02.004 21459050 PMC3084345

[bib42] Liberi G. Chiolo I. Pellicioli A. Lopes M. Plevani P., 2000 Srs2 DNA helicase is involved in checkpoint response and its regulation requires a functional Mec1-dependent pathway and Cdk1 activity. EMBO J. 19(18): 5027–5038. 10.1093/emboj/19.18.5027 10990466 PMC314228

[bib43] Liefshitz B. Parket A. Maya R. Kupiec M., 1995 The role of DNA repair genes in recombination between repeated sequences in yeast. Genetics 140: 1199–1211. 7498763 10.1093/genetics/140.4.1199 PMC1206687

[bib44] Liefshitz B. Steinlauf R. Friedl A. Eckardt-Schupp F. Kupiec M., 1998 Genetic interactions between mutants of the ‘error-prone’ repair group of Saccharomyces cerevisiae and their effect on recombination and mutagenesis. Mutat. Res. 407(2): 135–145. 10.1016/S0921-8777(97)00070-0 9637242

[bib45] Lin S. J. O’Connell M. J., 2017 DNA Topoisomerase II modulates acetyl-regulation of cohesin-mediated chromosome dynamics. Curr. Genet. 63(5): 923–930. 10.1007/s00294-017-0691-x 28382430 PMC5628089

[bib46] Liu J. Ede C. Wright W. D. Gore S. K. Jenkins S. S., 2017 Srs2 promotes synthesis-dependent strand annealing by disrupting DNA polymerase δ-extending D-loops. eLife 6: e22195. 28535142 10.7554/eLife.22195 PMC5441872

[bib47] Lue N. F. Yu E. Y., 2017 Telomere recombination pathways: tales of several unhappy marriages. Curr. Genet. 63(3): 401–409. 10.1007/s00294-016-0653-8 27666406 PMC5366096

[bib48] Machín F. Quevedo O. Ramos-Perez C. Garcia-Luis J., 2016 Cdc14 phosphatase: warning, no delay allowed for chromosome segregation! Curr. Genet. 62(1): 7–13. 10.1007/s00294-015-0502-1 26116076 PMC4723626

[bib49] Majka J. Burgers P. M., 2003 Yeast Rad17/Mec3/Ddc1: a sliding clamp for the DNA damage checkpoint. Proc. Natl. Acad. Sci. USA 100(5): 2249–2254. 10.1073/pnas.0437148100 12604797 PMC151326

[bib50] Marini V. Krejci L., 2012 Unwinding of synthetic replication and recombination substrates by Srs2. DNA Repair (Amst.) 11(10): 789–798. 10.1016/j.dnarep.2012.05.007 22921573 PMC3484393

[bib51] Miura T. Shibata T. Kusano K., 2013 Putative antirecombinase Srs2 DNA helicase promotes noncrossover homologous recombination avoiding loss of heterozygosity. Proc. Natl. Acad. Sci. USA 110(40): 16067–16072. 10.1073/pnas.1303111110 24043837 PMC3791737

[bib52] Mortensen U. H. Bendixen C. Sunjevaric I. Rothstein R., 1996 DNA strand annealing is promoted by the yeast Rad52 protein. Proc. Natl. Acad. Sci. USA 93(20): 10729–10734. 10.1073/pnas.93.20.10729 8855248 PMC38223

[bib81] Nagaraj V. H. O'Flanagan R. A. Bruning A. R. Mathias J. R. Vershon A. K., 2004 Combined analysis of expression data and transcription factor binding sites in the yeast genome. BMC Genomics 26: 5(1):59. 10.1186/1471-2164-5-59 PMC517709 15331021

[bib53] Palou R. Palou G. Quintana D. G., 2017 A role for the spindle assembly checkpoint in the DNA damage response. Curr. Genet. 63(2): 275–280. 10.1007/s00294-016-0634-y 27488803 PMC5383677

[bib54] Pannunzio N. R. Manthey G. M. Bailis A. M., 2008 RAD59 is required for efficient repair of simultaneous double-strand breaks resulting in translocations in Saccharomyces cerevisiae. DNA Repair (Amst.) 7(5): 788–800. 10.1016/j.dnarep.2008.02.003 18373960 PMC2422859

[bib55] Papouli E. Chen S. Davies A. A. Huttner D. Krejci L., 2005 Crosstalk between SUMO and ubiquitin on PCNA is mediated by recruitment of the helicase Srs2p. Mol. Cell 19(1): 123–133. 10.1016/j.molcel.2005.06.001 15989970

[bib56] Petukhova G. Stratton S. A. Sung P., 1999 Single strand DNA binding and annealing activities in the yeast recombination factor Rad59. J. Biol. Chem. 274(48): 33839–33842. 10.1074/jbc.274.48.33839 10567339

[bib57] Pfander B. Moldovan G. L. Sacher M. Hoege C. Jentsch S., 2005 SUMO-modified PCNA recruits Srs2 to prevent recombination during S phase. Nature 436(7049): 428–433. 10.1038/nature03665 15931174

[bib58] Prakash L., 1981 Characterization of postreplication repair in Saccharomyces cerevisiae and effects of rad6, rad18, rev3 and rad52 mutations. Mol. Gen. Genet. 184(3): 471–478. 10.1007/BF00352525 7038396

[bib59] Robert T. Dervins D. Fabre F. Gangloff S., 2006 Mrc1 and Srs2 are major actors in the regulation of spontaneous crossover. EMBO J. 25(12): 2837–2846. 10.1038/sj.emboj.7601158 16724109 PMC1500851

[bib60] Ruiz J. F. Gomez-Gonzalez B. Aguilera A., 2009 Chromosomal translocations caused by either pol32-dependent or pol32-independent triparental break-induced replication. Mol. Cell. Biol. 29(20): 5441–5454. 10.1128/MCB.00256-09 19651902 PMC2756893

[bib61] San Filippo J. Sung P. Klein H., 2008 Mechanism of eukaryotic homologous recombination. Annu. Rev. Biochem. 77(1): 229–257. 10.1146/annurev.biochem.77.061306.125255 18275380

[bib62] Saponaro M. Callahan D. Zheng X. Krejci L. Haber J. E., 2010 Cdk1 targets Srs2 to complete synthesis-dependent strand annealing and to promote recombinational repair. PLoS Genet. 6(2): e1000858 10.1371/journal.pgen.1000858 20195513 PMC2829061

[bib63] Schiestl R. H. Prakash S. Prakash L., 1990 The SRS2 suppressor of rad6 mutations of Saccharomyces cerevisiae acts by channeling DNA lesions into the RAD52 DNA repair pathway. Genetics 124: 817–831. 2182387 10.1093/genetics/124.4.817 PMC1203974

[bib64] Shinohara A. Shinohara M. Ohta T. Matsuda S. Ogawa T., 1998 Rad52 forms ring structures and co-operates with RPA in single-strand DNA annealing. Genes Cells 3(3): 145–156. 10.1046/j.1365-2443.1998.00176.x 9619627

[bib65] Singh S. Shemesh K. Liefshitz B. Kupiec M., 2013 Genetic and physical interactions between the yeast ELG1 gene and orthologs of the Fanconi anemia pathway. Cell Cycle 12: 1625–1636. 23624835 10.4161/cc.24756 PMC3680542

[bib66] Sugawara N. Ira G. Haber J. E., 2000 DNA length dependence of the single-strand annealing pathway and the role of Saccharomyces cerevisiae RAD59 in double-strand break repair. Mol. Cell. Biol. 20(14): 5300–5309. 10.1128/MCB.20.14.5300-5309.2000 10866686 PMC85979

[bib67] Symington L. S. Gautier J., 2011 Double-strand break end resection and repair pathway choice. Annu. Rev. Genet. 45(1): 247–271. 10.1146/annurev-genet-110410-132435 21910633

[bib68] Toh G. W. Lowndes N. F., 2003 Role of the Saccharomyces cerevisiae Rad9 protein in sensing and responding to DNA damage. Biochem. Soc. Trans. 31(1): 242–246. 10.1042/bst0310242 12546694

[bib69] Tsaponina O. Haber J. E., 2014 Frequent Interchromosomal Template Switches during Gene Conversion in S. cerevisiae. Mol. Cell 55(4): 615–625. 10.1016/j.molcel.2014.06.025 25066232 PMC4150392

[bib70] Ubersax J. A. Woodbury E. L. Quang P. N. Paraz M. Blethrow J. D., 2003 Targets of the cyclin-dependent kinase Cdk1. Nature 425(6960): 859–864. 10.1038/nature02062 14574415

[bib71] Valencia-Burton M. Oki M. Johnson J. Seier T. A. Kamakaka R., 2006 Different mating-type-regulated genes affect the DNA repair defects of Saccharomyces RAD51, RAD52 and RAD55 mutants. Genetics 174(1): 41–55. 10.1534/genetics.106.058685 16782999 PMC1569815

[bib72] Valencia M. Bentele M. Vaze M. B. Herrmann G. Kraus E., 2001 NEJ1 controls non-homologous end joining in Saccharomyces cerevisiae. Nature 414(6864): 666–669. 10.1038/414666a 11740566

[bib73] Van Komen S. Reddy M. S. Krejci L. Klein H. Sung P., 2003 ATPase and DNA helicase activities of the Saccharomyces cerevisiae anti-recombinase Srs2. J. Biol. Chem. 278(45): 44331–44337. 10.1074/jbc.M307256200 12966095

[bib74] Vaze M. B. Pellicioli A. Lee S. E. Ira G. Liberi G., 2002 Recovery from checkpoint-mediated arrest after repair of a double-strand break requires Srs2 helicase. Mol. Cell 10(2): 373–385. 10.1016/S1097-2765(02)00593-2 12191482

[bib75] Veaute X. Jeusset J. Soustelle C. Kowalczykowski S. C. Le Cam E., 2003 The Srs2 helicase prevents recombination by disrupting Rad51 nucleoprotein filaments. Nature 423(6937): 309–312. 10.1038/nature01585 12748645

[bib76] Weinert T. A. Hartwell L. H., 1988 The RAD9 gene controls the cell cycle response to DNA damage in Saccharomyces cerevisiae. Science 241(4863): 317–322. 10.1126/science.3291120 3291120

[bib77] Wright W. D. Heyer W. D., 2014 Rad54 functions as a heteroduplex DNA pump modulated by its DNA substrates and Rad51 during D loop formation. Mol. Cell 53(3): 420–432. 10.1016/j.molcel.2013.12.027 24486020 PMC4059524

[bib78] Wu Y. Sugiyama T. Kowalczykowski S. C., 2006 DNA annealing mediated by Rad52 and Rad59 proteins. J. Biol. Chem. 281(22): 15441–15449. 10.1074/jbc.M601827200 16565518

[bib79] Zhang H. Lawrence C. W., 2005 The error-free component of the RAD6/RAD18 DNA damage tolerance pathway of budding yeast employs sister-strand recombination. Proc. Natl. Acad. Sci. USA 102(44): 15954–15959. 10.1073/pnas.0504586102 16247017 PMC1276054

