# Supplementary figures and images for: Tight Regulation of Srs2 Helicase Activity Is Crucial for Proper Functioning of DNA Repair Mechanisms
Source: G3 (Bethesda). 2018 Mar 12;8(5):1615–26. doi: 10.1534/g3.118.200181 (PMC5940153; doi:10.1534/g3.118.200181)

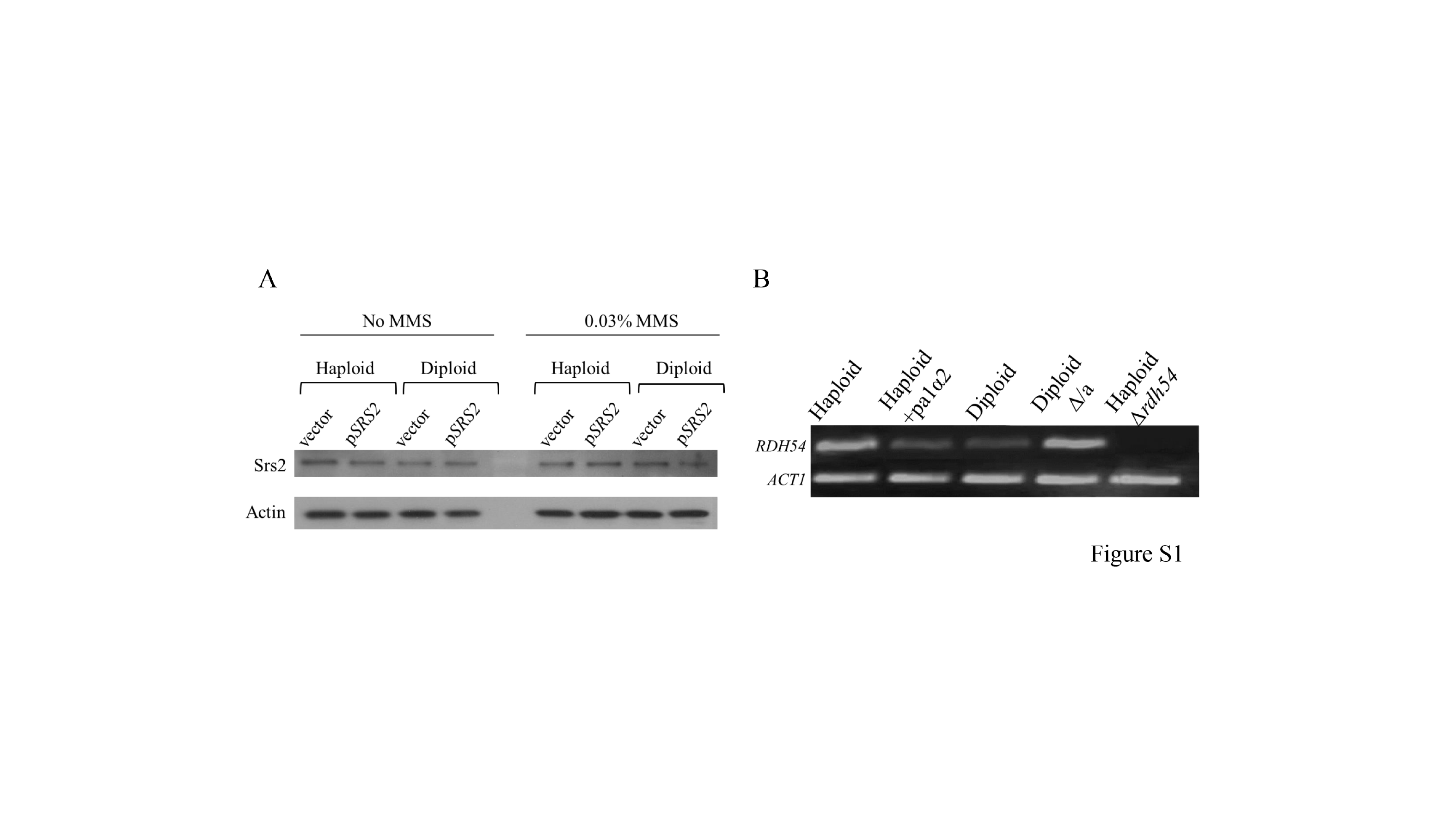

Supplement: Supplementary file 1 [file 1615FigureS1.tif]

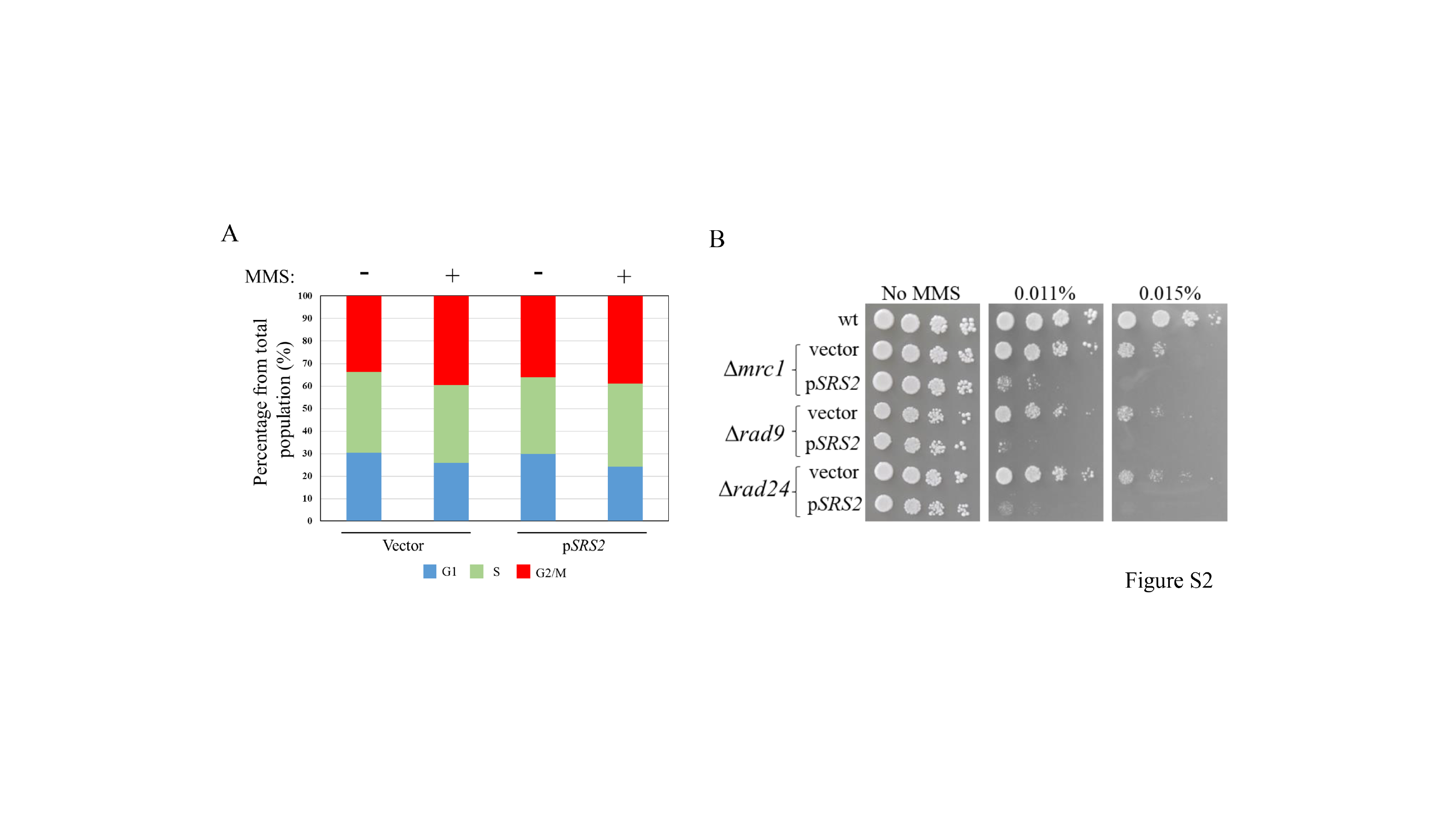

Supplement: Supplementary file 2 [file 1615FigureS2.tif]

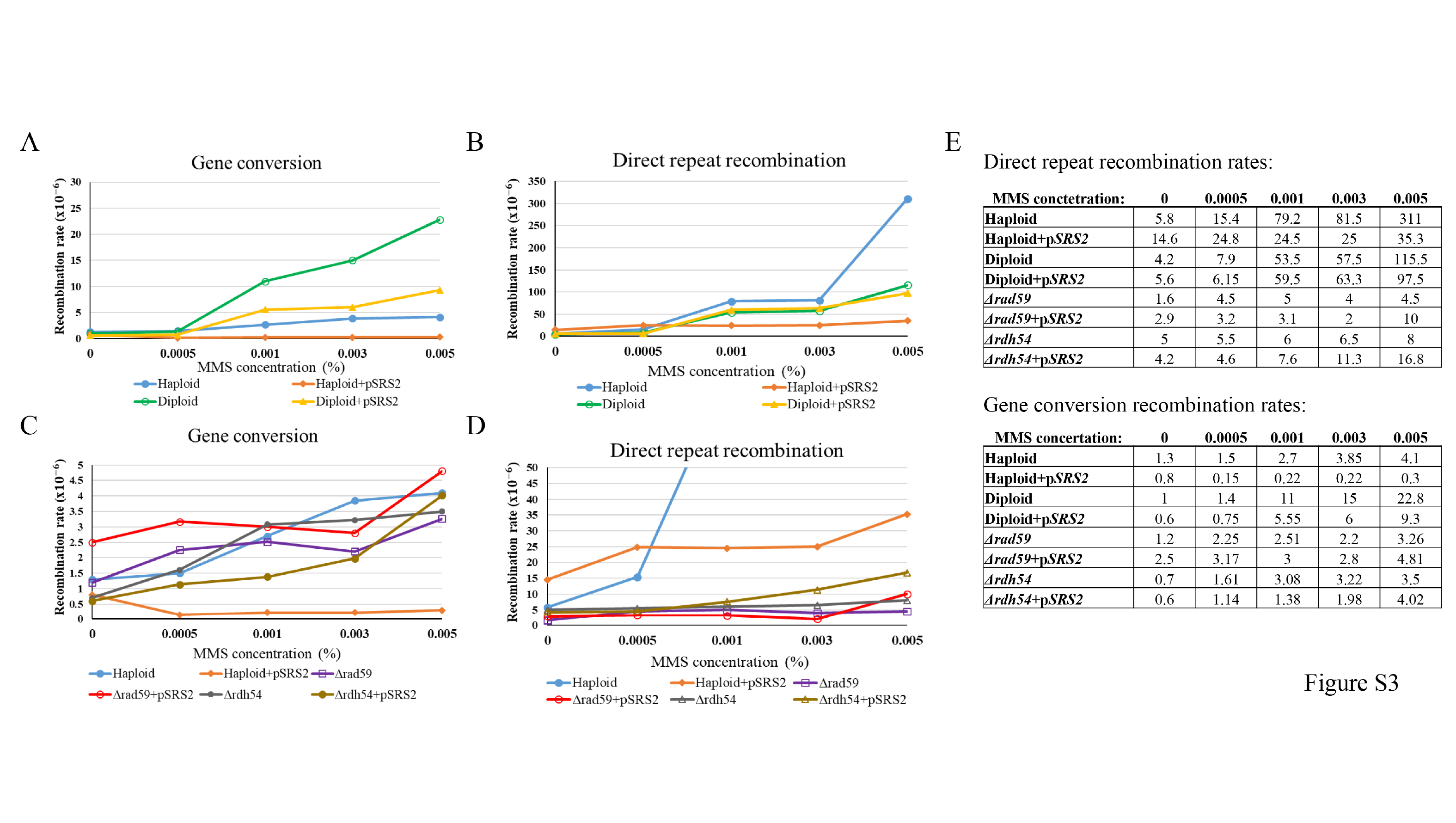

Supplement: Supplementary file 3 [file 1615FigureS3.tif]
